# Extended *RAS* and *BRAF* Mutation Analysis Using Next-Generation Sequencing

**DOI:** 10.1371/journal.pone.0121891

**Published:** 2015-05-08

**Authors:** Kazuko Sakai, Junji Tsurutani, Takeharu Yamanaka, Azusa Yoneshige, Akihiko Ito, Yosuke Togashi, Marco A. De Velasco, Masato Terashima, Yoshihiko Fujita, Shuta Tomida, Takao Tamura, Kazuhiko Nakagawa, Kazuto Nishio

**Affiliations:** 1 Department of Genome Biology, Kinki University Faculty of Medicine, Osaka-Sayama, Osaka, Japan; 2 Department of Medical Oncology, Kinki University Faculty of Medicine, Osaka-Sayama, Osaka, Japan; 3 Department of Biostatistics, Yokohama City University, Yokohama, Kanagawa, Japan; 4 Department of Pathology, Kinki University Faculty of Medicine, Osaka-Sayama, Osaka, Japan; University of Texas MD Anderson Cancer Center, UNITED STATES

## Abstract

Somatic mutations in *KRAS*, *NRAS*, and *BRAF* genes are related to resistance to anti-EGFR antibodies in colorectal cancer. We have established an extended *RAS* and *BRAF* mutation assay using a next-generation sequencer to analyze these mutations. Multiplexed deep sequencing was performed to detect somatic mutations within *KRAS*, *NRAS*, and *BRAF*, including minor mutated components. We first validated the technical performance of the multiplexed deep sequencing using 10 normal DNA and 20 formalin-fixed, paraffin-embedded (FFPE) tumor samples. To demonstrate the potential clinical utility of our assay, we profiled 100 FFPE tumor samples and 15 plasma samples obtained from colorectal cancer patients. We used a variant calling approach based on a Poisson distribution. The distribution of the mutation-positive population was hypothesized to follow a Poisson distribution, and a mutation-positive status was defined as a value greater than the significance level of the error rate (α = 2 x 10^-5^). The cut-off value was determined to be the average error rate plus 7 standard deviations. Mutation analysis of 100 clinical FFPE tumor specimens was performed without any invalid cases. Mutations were detected at a frequency of 59% (59/100). *KRAS* mutation concordance between this assay and Scorpion-ARMS was 92% (92/100). DNA obtained from 15 plasma samples was also analyzed. *KRAS* and *BRAF* mutations were identified in both the plasma and tissue samples of 6 patients. The genetic screening assay using next-generation sequencer was validated for the detection of clinically relevant *RAS* and *BRAF* mutations using FFPE and liquid samples.

## Introduction

Colorectal cancer is one of the leading causes of deaths due to cancer. As improved understanding of the molecular pathology of colorectal cancer, a number of targeted agents have been developed which have demonstrated improved outcome in colorectal cancer patients [1–6]. The signaling pathway of epidermal growth factor receptor (EGFR) plays a central role for the biology of colorectal cancer because two monoclonal antibodies directed to EGFR (cetuximab and panitumumab) have become important tools in the management of advanced disease [2, 3, 5].

KRAS is GTP-binding protein that is activated by GTP binding caused by upstream signals, such as EGFR. Single base mutations in the *KRAS* gene decrease GTPase activity, resulting in the constitutive activation of *KRAS*. The most common *KRAS* mutations are found in codons 12 and 13. Mutations in codons 12 and 13 of *KRAS* occur in ~40% of metastatic colorectal cancers [7, 8]. The OPUS and CRYSTAL studies of colorectal cancer patients receiving the FOLFOX plus cetuximab and FOLFIRI plus cetuximab regimens, respectively, have demonstrated that mutations in codons 12 and 13 are negative predictive factor for the response to the anti-EGFR antibody cetuximab [9, 10]. The addition of cetuximab improves survival only in patients with *KARS* wild-type colorectal cancer. Other studies have also shown that the *KRAS* mutation status was negatively correlated with the response to the treatment with anti-EGFR antibodies such as cetuximab and panitumumab [11–13].

*NRAS* is closely related to *KRAS*, having 85% amino acid sequence identity [14]. Recent studies showed that other activating mutations in *KRAS* (exons 3 and 4) or *NRAS* (exons 2, 3 and 4), in addition to *KRAS* mutation in exon 2 are also associated with poor prognosis or resistance to anti-EGFR antibody in metastatic colorectal cancer. The PRIME study showed that not only *KRAS*, but also *NRAS* mutations, will predict a lack of response to panitumumab [13]. Similar results were presented for extended RAS analyzes in the CRYSTAL and OPUS trials [15].

BRAF, a member of the Raf family of serine/threonine kinases, is a direct downstream effector of KRAS. *BRAF* mutation is constitutive active and leads to constitutive activation of mitogen-activated protein kinases (MAPK) pathway. The most common *BRAF* mutation is a T to A transversion resulting in a valine to glutamic acid substitution (V600E), present in ~10% of metastatic colorectal cancer patients [16]. *KRAS* and *BRAF* mutations are considered to be mutually exclusive [17]. In a retrospective analysis from the CRYSTAL and OPUS trials, the patients who had *BRAF* mutations had a poor prognosis, regardless of receiving chemotherapy or chemotherapy plus cetuximab [15].

These prospective-retrospective analyses have concluded that genetic testing for *KRAS*, *NRAS* and *BRAF* gene mutations beyond the routine analysis of *KRAS* exon 2 will be important for the selection of anti-EGFR therapy. Nevertheless, an efficient assay system for analyzing extended *RAS* and *BRAF* mutations in addition to those of exon 2 of *KRAS* (codons 12 and 13) is yet to be established. Many experimental methods can be used to detect rare mutations, such as mutation-specific PCR-based assays and high-resolution melting analysis. One disadvantage of these assays is that they consist of a single-plex reaction. Next-generation sequencing (NGS) is a widely used technology for gene mutation analysis. NGS technologies have the potential to analyze mutations in several genes and from multiple patient samples simultaneously. However, the ability of NGS technology to detect low-level mutations has limited its clinical use. In this study, we established and validated a gene mutation assay for the detection of *KRAS*, *NRAS*, and *BRAF* mutations in FFPE and plasma samples using an Ion Torrent PGM semiconductor sequencer (Life Technologies, Carlsbad, CA).

## Materials and Methods

### Ethics statement

This study was approved by the ethics committee of Kinki University Faculty of Medicine (Authorization Number: 25–015). All patients enrolled in the study provided written informed consent for the use of resected tissue.

### Samples

Using archived samples, 120 formalin-fixed, paraffin-embedded (FFPE) tissues from Japanese patients with primary colorectal cancer were examined. Of the 120 samples, 20 were used for the determination of cut-off value in the NGS assay, and the remaining 100 samples were used for mutation profiling. Matched plasma and FFPE samples were available for analysis from 15 patients with primary colorectal cancer. Normal blood samples were obtained from 10 healthy volunteers. Human non-small cell lung cancer cells (A549 and H1299) and human colorectal cancer cells (HCT116 and HT-29) were obtained from the American Type Culture Collection (Manassas, VA).

### DNA purification

Collected FFPE specimens were subjected to a histological review; only specimens containing sufficient tumor cells (at least 50% tumor cells) as determined by hematoxylin and eosin staining were subjected to DNA extraction. DNA was purified from FFPE specimens using a QIAamp DNA FFPE Tissue Kit (Qiagen, Valencia, CA) according to the manufacturer’s instructions. The total nucleic acid content was purified from 0.5 to 1.0 mL of plasma using a QIAamp Circulating Nucleic Acid Kit (Qiagen) according to the manufacturer's instructions. For the sensitivity assays, the cellular genomic DNA was extracted from four cultured cell lines: A549, H1299, HCT116, and HT-29. DNA was purified from the cell lines using a QIAamp DNA Mini Kit (Qiagen), according to the manufacturer’s instructions. Normal genomic DNA was also purified from blood obtained from 10 healthy human blood samples. The quality and quantity of the DNA were verified using the NanoDrop 2000 device (Thermo Scientific Wilmington, DE) and the PicoGreen dsDNA assay kit (Life Technologies). The extracted DNA was stored at 4°C until analysis.

### Mutation analysis using next-generation sequencing

The NGS assay for extended *RAS* and *BRAF* mutations was designed to detect mutations in codons 12, 13, 59, 61, 117, and 146 of *KRAS* and *NRAS* and in codon 600 of *BRAF* using an Ion Torrent PGM semiconductor sequencer (Life Technologies). The PCR primers were designed using Assay Designer 3.1 (Sequenom, San Diego, CA). Multiplex PCR was performed in a volume of 20 μL containing 10 ng of DNA, 4 U of Taq polymerase (Sequenom), 500 μM of each deoxynucleoside triphosphate (Sequenom), and 0.2 μM of each PCR primer. Thermal cycling was performed at 95°C for 15 min, followed by 45 cycles of 94°C for 20 sec, 56°C for 30 sec, and 72°C for 60 sec, followed by a final extension of 72°C for 3 min. The PCR products were purified using Agencourt AMPure XP beads (Beckman Coulter, Brea, CA), followed by the preparation of a barcoded DNA library using the Ion plus Fragment Library Kit (Life Technologies) and IonXpress Barcode Adaptors (Life Technologies). The quantified DNA libraries were pooled and sequenced using the Ion PGM 200 Sequencing Kit v2 and the Ion 318 v2 Chip Kit. The DNA sequencing data were accessed through the Torrent Suite v.4.0 software program. The reads were aligned against the hg19 human reference genome, and the variants were called using the plug-in Variant Caller (ver.4.0, Life Technologies).

### Data analysis of next-generation sequencing

To judge the presence of the mutant allele, the nucleotide count for each target base position was used from each allele count file in the variant calling report. Poisson distribution statistics were used to determine the presence of mutant nucleotides. The nucleotide count for each base position was applied to the Poisson function using Microsoft Excel 2013. The value of the baseline error rate (ER: non-reference nucleotide count divided by the total nucleotide count) plus its standard deviation (SD) calculated from 10 normal DNA samples was used for the Poisson parameter lambda (λ). When the nucleotide count of the mutant was significantly higher (α = 2 X 10^–5^) than that of the Poisson probability, the nucleotide was judged to have a mutation-positive status. We established the α value referring to the previous report [18].

### **Scorpion-AR**M**S**

Scorpion-ARMS real-time PCR (TheraScreen KRAS mutation kit, Qiagen) was used according to the manufacturer’s instructions. This assay is designed to detect a wild-type control and seven *KRAS* exon 2 mutations: *G12A*, *G12D*, *G12R*, *G12C*, *G12S*, *G12V*, and *G13D*. Real-time PCR was performed using the Rotor-Gene Q instrument (Qiagen). Data regarding each mutation was interpreted according to the kit manual after a curve analysis and the calculation of the δCt values (sample mutation assay Ct minus sample control assay Ct). The manufacturer has reported the sensitivity to be 1% mutant alleles in a wild-type background if a sufficient DNA input is used.

### Digital PCR

The sensitivity of the mutation analysis was determined using serial dilutions of extracted DNA from mutant-positive cultured cell lines. The accurate mutant allele frequency of the diluent was measured using the QX100 Droplet Digital PCR System according to the manufacturer’s instructions (Bio-Rad, Hercules, CA). The primers and probes for *KRAS G12A*, *G12C*, *G12D*, *G12R*, *G12S*, *G12V*, *G13D*, *NRAS Q61K*, and *BRAF V600E* were purchased from Bio-Rad. The PCR reaction was performed using the following cycling conditions: 95°C for 10 min, 40 cycles of 94°C for 30 sec and 55°C for 60 sec, followed by enzyme deactivation at 98°C for 10 min. After thermal cycling, the plates were transferred to a Droplet reader (Bio-Rad) to read the droplets. The digital PCR data was analyzed using QuantaSoft analysis software (Bio-Rad).

### Sequencing analysis

Sanger sequencing was performed for two sites (*KRAS 37G*, *175G* and *NRAS 351G*) with a high error rate (>10%) when examined using IonPGM sequencing. The primers for the PCR amplification and sequencing were as follows: *KRAS 37G*, 5’-AAGGCCTGCTGAAAATGACTG-3’ (forward) and 5’-GTCCTGCACCAGTAATATGC-3’ (reverse); *KRAS 175G*, 5’-GCACTGTAATAATCCAGACTG-3’ (forward) and 5’-CAATTTAAACCCACCTATAATGGT-3’ (reverse); and *NRAS 351G*, 5’- TTCCCGTTTTTAGGGAGCAG-3’ (forward) and 5’-GTCTGGTCTTGGCTGAGGTT-3’ (reverse). The purified products were sequenced using BigDye terminator v3.1 (Applied Biosystems, Foster City, CA) with the ABI 3100 Genetic Analyzer (Applied Biosystems).

### Statistical analysis

Kappa statistics were used to compare the NGS assay results with the results of the Scorpion-ARMS evaluation.

## Results

### Establishment of NGS assay to detect *KRAS*, *NRAS*, and *BRAF* mutations

**Fig 1:**
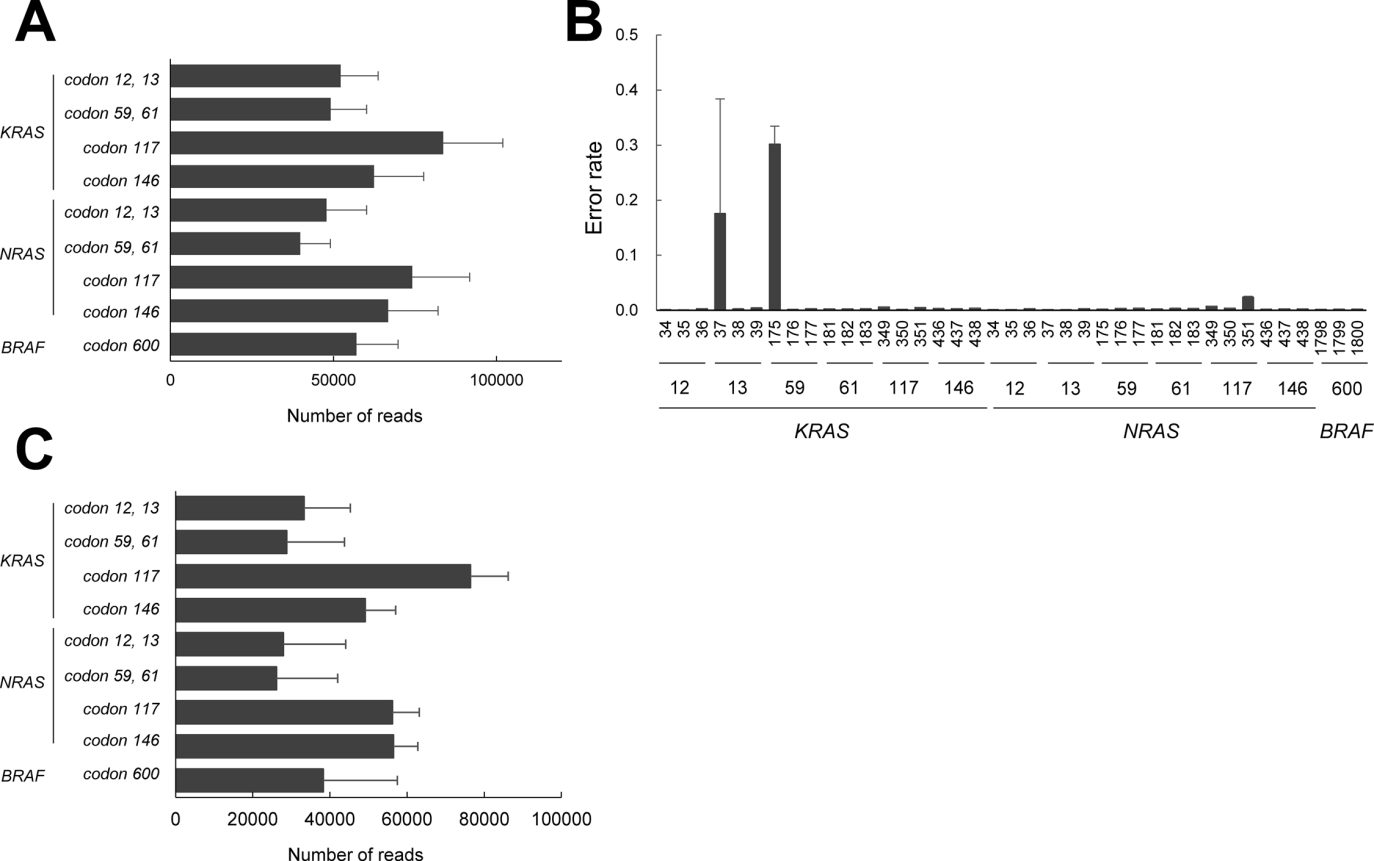
Read number and error rate of the NGS assay. (A) Average read number of 9 amplicons in 10 normal DNA samples (average with standard deviation). Horizontal axis, number of reads; vertical axis, PCR amplicon. (B) Error rate of position detection in 10 normal DNA samples (average with standard deviation). Horizontal axis, base position; vertical axis, error rate. (C) Average read number of 9 amplicons in 20 colorectal cancer FFPE DNA samples (average with standard deviation). Horizontal axis, number of reads; vertical axis, PCR amplicon.

This assay was designed to detect mutations at 13 codons in the *KRAS*, *NRAS*, and *BRAF* genes. Specific PCR primers were designed for exon 2 (codons 12 and 13), exon 3 (codons 59 and 61), and exon 4 (codons 117 and 146) of the *KRAS* and *NRAS* genes and exon 15 (codon 600) of the *BRAF* gene. Deep sequencing of a multiplex PCR-amplified fragment was performed to detect mutant alleles. The fragment was then read using a deep sequencer and was used to calculate the number of non-reference and reference nucleotides at the mutation sites. To check the read number and the sequencing error of the assay, we used normal DNA obtained from 10 healthy volunteers. The read number of each amplicon was calculated, and the median read number was 57,070 (range, 39,769 to 83,633) (Fig 1A).

The error rate (measured as the count of a non-reference nucleotide divided by the total sequencing depth at that position) for each position was also calculated. The error rates of most of the target sequences were below 0.01 (range; 0.0007–0.0071) except for three mutation sites: *KRAS 37G* (0.1755), *175G* (0.3016), and *NRAS 351G* (0.0233). A high error rate of G>A substitution was detected at these three sites, compared with that at other mutation sites (Fig 1B). We re-analyzed the sequences at these three sites using Sanger sequencing and found that the errors occurred during the PCR or sequencing process. Because of the high rates of read errors at these three sites, the sites were omitted from the evaluation.

### Determination of criteria for mutant detection

**Fig 2:**
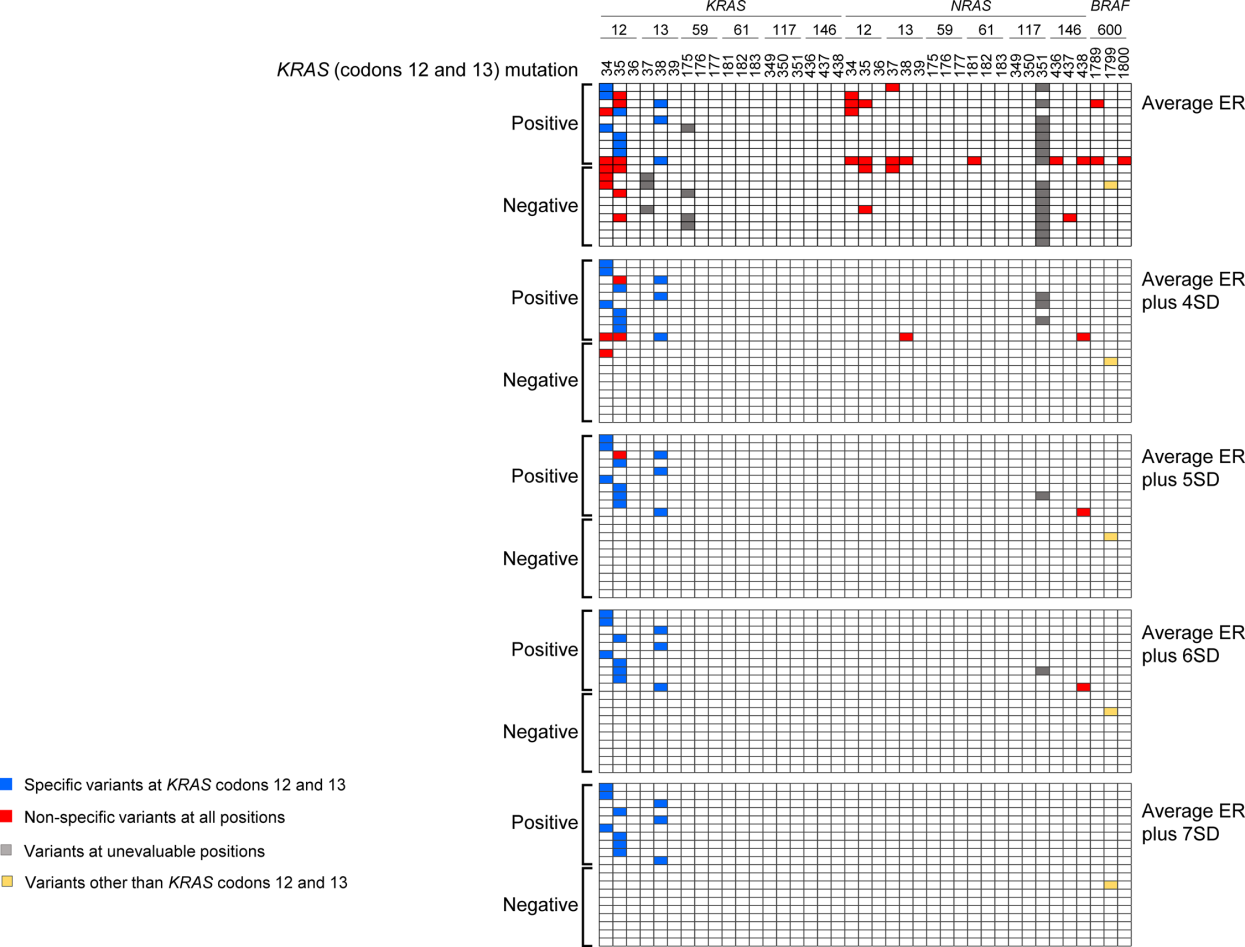
Adjustment of Poisson coefficient as cut-off value. Results of mutation analysis in 20 FFPE samples when different Poisson coefficients (average ER to average ER plus 7SD) were used. Horizontal axis, base position; vertical axis, 20 FFPE samples from colorectal cancer patients (10 *KRAS* exon 2 mutation-positive and 10 mutation-negative samples). Blue, specific variant detection at *KRAS* codons 12 and 13; Red, non-specific variant detection at all positions; Gray, variant detection at unevaluable positions; Yellow, variants other than *KRAS* codons 12 and 13.

The Poisson distribution model is commonly used to detect rare events. We applied the read error rate to the Poisson parameter lambda (λ) and calculated the Poisson probability. The number of nucleotides at a base position in each sample was regarded as a Poisson distribution. When the calculated Poisson probability was higher than that of the λ value at a significance level of *α* = 2 x 10^–5^, the sample was judged to have a mutation-positive status. The mutant detection limit (number of minimum required non-reference nucleotides) was dependent on the λ value. The error rate at each position was used as the λ value to determine the Poisson probability. To optimize the λ value to determine the mutant detection criteria, we used 20 colorectal cancer FFPE samples (10 *KRAS* exon 2 mutation-positive and 10 mutation-negative samples). The median read number of 9 amplicons in 20 FFPE samples was 38,252 (range; 26,163–76,420) and was comparable to that of normal samples (Fig 1C). We applied the average error rate (ER) plus various ranges of the standard deviation (SD; 1SD— 7SD) as the λ value. When the average ER plus 5SD was used, the *KRAS* exon 2 mutation-negative samples were correctly identified as “negative” (Fig 2).

When the average ER plus 7SD was used, the *KRAS* exon 2 mutation-positive samples were correctly identified as “positive” (Fig 2). Based on these results, the average ER plus 7SD was used as the λ value in the subsequent analyses. The *BRAF V600E* mutation was detected in a *KRAS* mutation-negative sample.

### Minimum detection limit

Next, we examined the minimum detection limit of the assay. Four cell lines with *KRAS G12S* (A549), *KRAS G13D* (HCT116), *NRAS Q61K* (H1299), and *BRAF V600E* (HT-29) were used. We performed a NGS assay of serially diluted DNA samples obtained from the cell lines and judged whether the samples were mutation-positive or mutation-negative based on the criterion described above. The rate of mutant DNA in each diluted sample was measured using digital PCR. The minimum detection limit of the NGS assay was determined to be 2.40% for *KRAS G12S*, 3.92% for *KRAS G13D*, 0.58% for *NRAS Q61K*, and 1.85% for *BRAF V600E* (Table 1).

**Table 1:** Minimum detection limit of the NGS mutation assay.

	Position (Amino acid)	Mutation rate (%)	NGS mutation assay
*KRAS*	c.34G>A (G12S)	12.31	Positive
		5.93	Positive
		2.40	Positive
		1.18	Negative
		0.66	Negative
		0.27	Negative
*KRAS*	c.38G>A (G13D)	11.66	Positive
		3.92	Positive
		2.30	Negative
		1.46	Negative
		0.50	Negative
		0.32	Negative
*NRAS*	c.181C>A (Q61K)	74.40	Positive
		5.19	Positive
		1.49	Positive
		0.58	Positive
		0.31	Negative
		0.07	Negative
*BRAF*	c.1798G>T (V600E)	8.19	Positive
		3.45	Positive
		1.85	Positive
		0.90	Negative
		0.52	Negative
		0.28	Negative

The minimum detectable mutant frequency is underlined.

Thus, the NGS assay was demonstrated to be sensitive enough and comparable to that of standard assays, such as Scorpion-ARMS.

### Clinical performance of extended *RAS* and *BRAF* mutation assay for FFPE samples

Using our NGS assay, we examined the mutation status of 100 FFPE tissue samples from patients with colorectal cancer who were treated at Kinki University Hospital. To verify the FFPE DNA, the PCR products were analyzed by using the Agilent 2100 Bioanalyzer. All PCR fragments were amplified successfully. A sufficient read number was obtained using sequencing. The median read number of 9 amplicons in 100 archived FFPE samples was 34,641 (range, 18,402 to 61,109). We identified *KRAS* mutations in 47/100 (47%), *NRAS* mutations in 7/100 (7%), and *BRAF* mutations in 5/100 (5%). The mutations detected in *KRAS* were mainly located in codons 12 and 13 (43/47, 91.4%). To determine the concordance of the assays, the results for the *KRAS* exon 2 mutations were compared with the results obtained using the Scorpion-ARMS assay and digital PCR assay. The data of Scorpion-Arms used to evaluate accordance was determined from the criteria in the kit. The data of digital PCR to be used for judging accordance was a decided on the following criteria. The cutoff values for digital PCR were determined by using the data of mutation-negative FFPE samples. The average and standard deviation for *KRAS G12A*, *G12C*, *G12D*, *G12R*, *G12S*, *G12V* and *G13D* mutant copy number in 9 FFPE samples was calculated. The average copy numbers of negative samples plus 3SD was used as the cutoff value for each mutation site; the higher value of either, the average plus 3SD or three copies was used as the cutoff value. The cutoff values were set at 3.00 copies for *KRAS G12A*, 3.10 copies for *KRAS G12C*, 15.27 copies for *KRAS G12D*, 3.00 copies for *KRAS G12R*, 16.48 copies for *KRAS G12S*, 4.32 copies for *KRAS G12V* and 18.75 copies for *KRAS G13D*, respectively.

Table 2 shows the concordance of the data between Scorpion-ARMS and NGS assay. Thirty-six mutations in codon 12 were detected using the NGS assay, but 10 of these mutations were not detected using Scorpion-ARMS. Eight mutations at codon 13 were identified in both of the assays. Three samples exhibited double-point mutations: in one sample, both assays detected *G12R* and *G12D* mutations, whereas in the other two samples, *G12R* and *G12D* or *G12S* and *G13D* was detected using only the NGS assay. The overall concordance of *KRAS* mutations in exon 2 as detected using the NGS assay and the Scorpion-ARMS was 92/100 (92%) (κ value: 0.833).

**Table 2:** Comparison of Scorpion-ARMS and NGS mutation assay results for *KRAS* mutations (exon 2) in archived FFPE tissues.

		Scorpion-ARMS		Total
		Positive	Negative	
NGS mutation assay	Positive	35	8	43
	Negative	0	57	57
Total		35	65	100

In comparing between digital PCR and NGS of the 100 samples, 36 mutations in codon 12 were detected using the NGS assay, but 9 of these mutations were not detected using digital PCR. Three mutations in codon 12 were detected by digital PCR but not detected by the NGS assay. Eight mutations at codon 13 were shared by both of the assays. The concordance of both assays has been shown in Table 3. Four samples exhibited double-point mutations. In one sample, *G12R* and *G12D* mutations were shared in both assay. In the other three samples, *G12R* and *G12D* and *G12S* and *G13D* or *G12C* and *G12D* were detected in either NGS assay or digital PCR, respectively. Then, the overall concordance of *KRAS* mutations in exon 2 detected by NGS assay and digital PCR was 90/100 (90%) (κ value: 0.794). Comparison with digital PCR assay, false-positive and false-negative rate of the NGS assay against digital PCR assay were 11.5% and 7.7%, respectively.

**Table 3:** Comparison of digital PCR and NGS mutation assay results for *KRAS* mutations (exon 2) in archived FFPE tissues.

		Digital PCR		Total
		Positive	Negative	
NGS mutation assay	Positive	36	7	43
	Negative	3	54	57
Total		39	61	100

In total, NGS assay showed high consistency with the Scorpion-ARMS assay (92%) and the digital PCR assay (90%) for *KRAS* mutations in exon 2. Therefore we concluded that the NGS assay is feasible for the detection of mutations using FFPE tissues.

### Feasibility test for cfDNA

To gauge the feasibility of using this assay for liquid samples, we used it to detect mutations in cell-free DNA (cfDNA). Fifteen FFPE tumor-tissues and 15 matched plasma samples were assessed. Of the 7 patients whose FFPE specimens exhibited *KRAS* mutations, these mutations were found in at least one of the plasma samples from 5 patients. No mutation was found in the plasma samples of the 4 patients whose FFPE specimen exhibited an *NRAS* mutation. The *BRAF V600E* mutation was found in both FFPE tumor and plasma specimens from one patient. All of the obtained data is shown in Table 4.

**Table 4:** Concordance of plasma and tumor results for *RAS* and *BRAF* mutations.

Sample	*KRAS*		*NRAS*		*BRAF*	
	Tumor	Plasma	Tumor	Plasma	Tumor	Plasma
DS1						
DS3	*G12S*, *G12D*		*G12S*			
DS4						
DS6	*G13D*	*G12S*, *G13D*				
DS8	*G12V*	*G12V*				
DS9	*G12S*, *A59E*		*G12S*, Q61*			
DS22					*V600E*	*V600E*
DS23						
DS24						
DS25	*G12S*	*G12S*				
DS28			*A146V*, *A146A*			
DS29			*G12S*, *G12D*,*Q61K*			
DS30	*G12S*	*G12S*				
DS32	*G12V*	*G12V*				
DS33						

## Discussion

Anti-EGFR therapy significantly improved clinical outcome in metastatic colorectal cancer, but no treatment benefit was seen in patients who had *KRAS* mutations in codons 12 and 13. In addition, other activating mutations in *RAS* (*KRAS* or *NRAS*) or *BRAF* have been suggested as negative predictive biomarkers for anti-EGFR therapy [11–13, 15]. Thus, genetic profiling of extended *RAS* and *BRAF* mutations is considered to be clinically important.

We have established a NGS mutation assay for the detection of *KRAS*, *NRAS*, and *BRAF* mutations in clinical FFPE samples of colorectal cancer and have demonstrated a high preclinical and clinical performance. The detection of *KRAS* mutations using deep sequencing has been reported in previous clinical studies, such as the 20020408 study [19]. However, the preclinical and clinical performance of an NGS system for the detection of gene sets including minor mutations in *KRAS*, *NRAS*, and *BRAF* in patients with colorectal cancer has not been previously reported.

In the NGS analysis, some problems with mutation calling were noted. The greatest concern was the frequency of errors introduced during the PCR amplification and sequencing processes. We estimated the error rates in 10 samples of normal DNA and observed high error rates at three positions. It is unclear why such a high incidence of G>A substitution occurred at these positions, but the overall error rate at the other positions was consistent with the results of a previous report [20]. To reduce the high error rate at the three positions, we compared both Taq polymerase (Sequenom) and high fidelity enzymes Platinum Taq DNA polymerase (Thermo Scientific). The amount of PCR product achieved by Taq polymerase was greater than Platinum Taq DNA polymerase. A high error rate of G>A substitution at *KRAS 37G*, *175G*, and *NRAS 351G* was also detected in using Platinum Taq DNA polymerase. Therefore we selected Taq polymerase for the NGS assay. In addition, PCR amplification in 9 amplicons was not uniform. This is probably due to the affinity differences of the primers. In the future, adjustment for each primer concentration in the multiplex PCR will be necessary in order to ensure uniform coverage.

The detection of rare mutations is affected by sequencing errors. To determine whether the non-reference allele counts were “mutation positive”, we adjusted the value of the average ER plus 7SD as the Poisson coefficient. The coefficient allowed us to distinguish properly between a mutation-positive status and a mutation-negative status in 20 FFPE tumor samples. When we used this cut-off value, a high concordance was obtained between the NGS assay and the Scorpion-ARMS assay. Therefore, our decision criterion for the mutation analysis was considered to be appropriate.

We examined the assay sensitivity of the NGS assay. The experimental sensitivity of our assay was estimated to be 0.58%– 3.92%. In a prospective-retrospective analysis of pivotal clinical studies, several kinds of *KRAS* mutation assays were used, including direct sequencing, SURVEYOR, pyrosequencing, and BEAMing [21–23]. The sensitivities of these assays were estimated to be 1% to 10%. Thus, the sensitivity of the NGS assay was sufficiently high for clinical use. The success rate of the NGS assay for clinical FFPE samples was 100% without an invalid case. High concordance was observed between this assay and the Scorpion-ARMS evaluation for the detection of mutations in *KRAS* exon 2 (codons 12 and 13), allowing us to speculate that this assay is a reliable means of detecting other mutation sites.

Various kinds of *KRAS*, *NRAS*, and *BRAF* mutations were detected in 100 FFPE samples. The *KRAS*, *NRAS*, and *BRAF* mutations were identified in 59/100 (59%) of the samples using the NGS mutation assay. The mutations detected in *KRAS* were mainly located in codons 12 and 13 (43/47, 91.4%). Other minor mutations (minor *KRAS*, *NRAS*, and *BFAF*) were detected in 16/100 cases (16%). The additional mutations in minor *KRAS*, *NRAS*, and *BRAF* were detected in 20% of the colorectal cancer patients in the PEAK study [24]. This detection rate of the NGS mutation assay was considered to be comparable to those of previous reports. There was no significant association between mutational status and clinicopathologic features (age, sex or historical subtype). This results are consistent with the previous reports [25, 26].

We also attempted to detect mutations in cfDNA obtained from plasma samples. The detection of *KRAS* mutations in cfDNA or circulating tumor cells has been reported to be clinically meaningful [27, 28]. These results are especially meaningful for the monitoring of treatment with molecular targeted agents, such as anti-EGFR antibodies for colorectal cancer and EGFR-tyrosine kinase inhibitors for lung cancer [29–31]. Several sensitive technologies, such as BEAMing, Scorpion-ARMS, and SABER, have been used to detect somatic mutations in cfDNA [22, 32, 33]. Several reports, including ours, have demonstrated variations in the concordance of results for tumors and cfDNA [32–35]. In the present study, *KRAS* and *BRAF* mutations were identified in 6/15 cases, and the concordance between the FFPE samples and the cfDNA in these patients was 70%. Discordance between the tumor DNA and the cfDNA was observed in four cases. This might be due to the quality of the FFPE derived DNA. Indeed, among the fifteen matched samples, the mutation status of relatively fresh 5 FFPE samples (obtained in 2013) was consistent with that of the respective plasma samples. On the other hand, among the five discordant cases (DS3, DS6, DS9, DS28, DS29), FFPE samples from four of these cases (DS3, DS9, DS28, DS29) were obtained from 2007 to 2011. Thus, we speculate that the degradation of DNA in older FFPE samples over time caused the discordance between FFPE and plasma samples. In addition, there is the possibility that *KRAS G12S* in DS6 might be false-positive mutation, although the discrepancy might be explained by heterogeneity. We performed digital PCR on these samples and detected no mutations in *KRAS G12S*. In comparing NGS and digital PCR assay using 100 FFPE samples, a high rate of discordance was observed in *KRAS G12S*. Therefore discordance in *KRAS G12S* in DS6 might be due to false-positive results by the NGS assay. Although the overall concordance between the NGS and digital PCR assay was 90%, we would like to improve the specificity of *KRAS G12S* in the near future.

In summary, we have confirmed the feasibility of the NGS assay for *KRAS*, *NRAS*, and *BRAF* mutation screening. Only 10 ng of input DNA was sufficient for a successful analysis. Our results indicate that screening for *KRAS/NRAS/BRAF* mutations using NGS is a practical method in FFPE and plasma samples. Future studies are needed to evaluate the significance of the assay in the clinical setting.

## Supporting Information

S1 Fig Schematic of the NGS assay. Sequencing reads were mapped to the respective gene followed by variant calling. The number of non-reference reads were statistically filtered with average error rate plus 7SD threshold.
(TIFF)

S2 Fig Gel image for PCR products. PCR products were separated with the use of an Agilent 2100 Bioanalyzer and a DNA 1000 LabChip Kit (Agilent Technologies, Inc., Palo Alto, CA). The PCR products are from 100 FFPE samples and 15 matched plasma and FFPE samples. The expected size of the amplification products were approximately 100 bp (indicated by an arrow). Several extra products were detected in addition to the expected product (150 bp or more).
(TIFF)

S1 Table PCR (F, forward; R, reverse) primers. (XLSX)

S2 Table Error rates for KRAS, NRAS, and BRAF gene mutations using the NGS assay. (XLSX)

S3 Table Patient characteristics (n = 100). (XLSX)

S4 Table Detected mutations in archived FFPE tissues using the NGS, Scorpion-ARMS and digital PCR assays. (XLSX)
